# Pharmacokinetic and pharmacodynamic properties of cannabigerol in male mice

**DOI:** 10.1016/j.jpet.2026.104308

**Published:** 2026-02-24

**Authors:** Alex Mabou Tagne, Faizy Ahmed, Adren Tran, Francesca Galvani, Lana Debbaneh, Emma Raine Perranoski, David Sarlah, Aditi Das, Elisa Pabon, Ziva Cooper, Daniele Piomelli

**Affiliations:** 1 Department of Anatomy and Neurobiology, University of California, Irvine, Irvine, California; 2 Department of Pharmaceutical Sciences, Jerry H. Hodge School of Pharmacy, Texas Tech University Health Science Center, Amarillo, Texas; 3 Department of Chemistry, Rice University, Houston, Texas; 4 School of Chemistry and Biochemistry, Georgia Institute of Technology, Atlanta, Georgia; 5 Jane and Terry Semel Institute for Neuroscience and Human Behavior, Department of Psychiatry and Biobehavioral Sciences, David Geffen School of Medicine, University of California, Los Angeles, Los Angeles, California; 6 Jane and Terry Semel Institute for Neuroscience and Human Behavior, Department of Psychiatry and Biobehavioral Sciences, Department of Anesthesiology and Perioperative Medicine, David Geffen School of Medicine, University of California, Los Angeles, Los Angeles, California; 7 Department of Biological Chemistry, University of California Irvine, Irvine, California; 8 Department of Pharmaceutical Sciences, University of California, Irvine, Irvine, California

**Keywords:** Cannabigerol, Cyclo-cannabigerol, Cannabis, Pharmacokinetics, Liquid chromatography/tandem mass spectrometry, Anxiety

## Abstract

Cannabigerol (CBG) is a nonintoxicating phytocannabinoid gaining popularity as a self-medication for anxiety and other conditions; however, its pharmacological properties remain poorly defined. Here, we report the development of a rapid and sensitive liquid chromatography–tandem mass spectrometry method for quantifying CBG and its primary oxidative metabolite, cyclo-CBG. This platform enabled the characterization of CBG’s pharmacokinetic and biotransformation profile after intraperitoneal administration (10 mg/kg) in male mice. CBG exhibited rapid systemic distribution and clearance, with relatively low brain penetration (brain-to-plasma ratio = 0.26). In contrast, cyclo-CBG accumulated in brain tissue to a surprising extent (brain-to-plasma ratio = 7.1), suggesting local formation and a potentially important role in mediating central effects. Despite prior reports of anxiolytic effects, we found that CBG administered at its peak brain concentration produced anxiogenic-like effects in mice, as assessed using the elevated plus maze. This response was not affected by the CB_1_ cannabinoid receptor inverse agonist, rimonabant (3 mg/kg, i.p.), indicating a mechanism independent of CB_1_ signaling. As interest in CBG continues to rise, the analytical and pharmacokinetic framework presented here provides a valuable foundation for advancing preclinical and clinical investigations into its efficacy, safety, and mechanism of action.

**Significance Statement:**

Application of a new liquid chromatography–tandem mass spectrometry method to quantify cannabigerol reveals key pharmacokinetic properties of this phytocannabinoid in mice, including unexpectedly high brain accumulation of its metabolite cyclo-cannabigerol, which was accompanied by anxiogenic-like effects. The results offer valuable tools for advancing preclinical and clinical investigations into cannabigerol pharmacology.

## Graphical abstract

**Figure:**
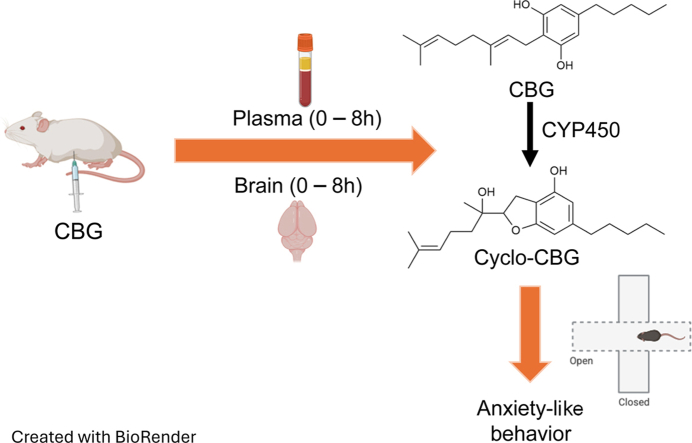

## 1 Introduction

Cannabigerol (CBG) is a nonintoxicating constituent of cannabis^1^ that, in its acidic form, serves as the biosynthetic precursor to the major phytocannabinoids Δ^9^-tetrahydrocannabinol and cannabidiol, but does not share their pharmacological properties.^2^ CBG is currently marketed—alone or in combination with other substances—as a dietary supplement and is reportedly used to self-medicate anxiety, chronic pain, depression, and insomnia.^3^ Despite being widely available to the public, CBG remains understudied and its pharmacodynamic properties are still incompletely characterized.^2^ Pharmacodynamic studies in mice and rats support a range of potential therapeutic benefits, including alleviation of anxiety,^4^^,^^5^ pain,^6^^,^^7^ bowel inflammation,^8^ and hypertension.^9^ These effects may involve the modulation of *α*_2_-adrenergic and 5-HT_1A_ receptors, as well as TRPA1 ion channels, to which CBG binds with subnanomolar to submicromolar affinity.^2^ Although fully controlled clinical studies are still lacking, a double-blind, placebo-controlled crossover field trial reported reduced anxiety in 34 healthy adults after a single oral dose of CBG.^10^ These promising findings require replication in larger, rigorously controlled cohorts.

A detailed understanding of CBG’s pharmacokinetics (PK) and biotransformation is an important prerequisite for its progression through preclinical and clinical development. Several studies have begun to characterize its absorption and distribution in mice and rats^11^^,^^12^ as well as in humans.^13^ Notably, a recent report identified cyclo-CBG as the primary first-pass metabolite generated by human cytochrome P450 enzymes.^14^ Although cyclo-CBG exhibits biological activity, modifying the function of brain microglia,^14^ it has been largely overlooked in the development of analytical methods and in prior PK investigations.^12^^,^^13^ Thus, validated assays capable of quantifying both CBG and this potentially important metabolite are still needed.

To address this knowledge gap, we developed a rapid, sensitive, and reproducible liquid chromatography–tandem mass spectrometry (LC-MS/MS) method for the simultaneous quantification of CBG and cyclo-CBG in mouse plasma and brain tissue. The method was optimized to overcome matrix effects linked to CBG’s hydrophobicity and improve analyte recovery in both aqueous and lipid-rich compartments. Using this platform, we characterized the PK profile of CBG in adult male mice and found that a 10 mg/kg (i.p.) dose produces pharmacologically relevant concentrations of both CBG and cyclo-CBG in brain tissue. To explore functional consequences, we evaluated the impact of CBG administration on anxiety-like behavior using the elevated plus maze (EPM), a well established assay for assessing innate fear in rodents.^15^ Contrary to previous reports of anxiolytic-like effects,^11^^,^^12^ we found that CBG produced anxiogenic-like behavior in this model. The response appeared to be independent of cannabinoid CB_1_-receptor signaling as it was not affected by coadministration of the CB_1_ inverse agonist rimonabant. The new analytical method and PK characterization provide a foundation for future studies aimed at elucidating the mechanisms, efficacy, and safety of CBG and its metabolites.

## 2 Materials and methods

### 2.1 Solvent and chemicals

CBG and internal standard [^2^H_9_]-CBG were obtained from Cayman Chemicals. Cyclo-CBG was synthesized in the laboratory of Dr David Sarlah (Rice University). LC/MS-grade water and methanol were from Honeywell. LC/MS-grade acetonitrile, isopropanol, rimonabant, and acetone were from Sigma-Aldrich. LC/MS-grade formic acid was from Thermo Fisher.

### 2.2 Animals

Male CD-1 mice (8 weeks of age upon arrival) were purchased from Charles River Laboratories. The animals were housed in groups of 5 per cage in rooms maintained on a 12-hour light/12-hour dark cycle (lights on at 6:30 am and off at 6:30 pm) under controlled temperature (20 ± 2 °C) and relative humidity (55%–60%). They had ad libitum access to food and water. All procedures were approved by the Institutional Animal Care and Use Committee at the University of California, Irvine (AUP-23-082), and carried out in strict accordance with the National Institutes of Health guidelines for care and use of experimental animals.

### 2.3 Standard preparation

Stock solutions of CBG, cyclo-CBG and [^2^H_9_]-CBG were prepared in methanol (1 *μ*g/mL). Serial methanol dilutions (from 1.0 *μ*g/mL to 0.02 ng/mL) were used to generate calibration curves for CBG and cyclo-CBG.

### 2.4 Equipment

Chromatographic separations were carried out using a 1260 series LC system (Agilent Technologies) consisting of a binary pump, degasser, temperature-controlled autosampler, and thermostated column compartment, coupled to a 6410B triple quadrupole mass spectrometric detector with electrospray ionization interface.

### 2.5 LC conditions

An Eclipse Plus C_18_ (1.8 *μ*m, 2.1 × 30 mm) analytical column, coupled with a Poroshell EC-C18 (1.8 *μ*m, 2.1 × 5.0 mm) guard column (Agilent Technologies), was used for LC separations. The mobile phase consisted of 0.1% formic acid in water (solvent A) and 0.1% formic acid in methanol (solvent B). The flow rate was maintained at 0.3 mL/min. An initial isocratic mobile phase consisting of 80% B was held for 2 minutes, the retention time window of both analytes, followed by an increase to 95% B from 2.01 minutes to 2.5. At 2.51 minutes, the gradient was returned to 80% B and maintained until 3.5 minutes at a flow rate of 0.4 mL/min for fast column re-equilibration. The column temperature was set to 45 °C, and the injection volume was 1.0 *μ*L. To minimize carryover, the needle was washed in the autosampler port for 10 seconds before each injection using a solution of 10% acetone in water/methanol/isopropanol/acetonitrile (1:1:1:1, v/v).

### 2.6 Mass spectrometric detector conditions

The mass spectrometric detector was operated in the positive electrospray ionization interface mode, and analytes were quantified using Multiple Reaction Monitoring with Multiple Reaction Monitoring transitions listed in Table 1. Acquisition parameters and source parameters were optimized for each analyte using Agilent MassHunter Optimizer software. The drying gas temperature was set at 300 °C, with flow rate of 9.0 L/min. Nebulizer pressure was maintained at 50 psi, whereas capillary voltages was set at 3000 V. The Agilent MassHunter software was used for instrument control, data acquisition and analysis.

**Table 1:** Mass spectrometry parameters for analytes under study

Analyte	Precursor ion (*m/z*)	Production (*m/z*)	Dwell Time (ms)	Fragmentation Voltage (V)	Collision Energy (V)	ESI Mode
CBG^a^	317.25	193.0	200	95	13	+
CBG^b^	317.25	122.9	200	95	33	+
Cyclo-CBG^a^	333.25	193.0	200	95	17	+
Cyclo-CBG^b^	333.25	122.9	200	95	37	+
[^2^H_9_]-CBG^a^	326.31	202.1	200	85	17	+
[^2^H_9_]-CBG^b^	326.31	122.9	200	85	37	+

ESI, electrospray ionization interface.

a Quantifier.

b Qualifier.

### 2.7 Analyte extraction

CBG and cyclo-CBG were extracted from tissues using a previously described method.^16^^,^^17^ Briefly, plasma (0.1 mL) was transferred into 8 mL glass vials (Thermo Fisher Scientific), and proteins were precipitated by adding 0.5 mL of ice-cold acetonitrile containing 1% formic acid and [^2^H_9_]-CBG internal standard (20 ng/mL). Frozen half brains were placed in 7 mL Precellys CK14 soft tissue homogenization tubes (Bertin Instruments) and homogenized in 5 mL of ice-cold acetonitrile with 1% formic acid and the same internal standard mixture. Plasma samples and brain homogenates were vortexed for 30 seconds and centrifuged at 1450 × *g* for 15 minutes at 4 °C. The resulting supernatants were loaded onto Enhanced Matrix Removal (EMR)-Lipid cartridges (Agilent Technologies) and eluted under low positive pressure (3–5 mm Hg, ∼1 drop/5 seconds). Residual pellets from both plasma and brain extractions were resuspended in a water:acetonitrile mixture (1:4, v/v, 0.2 mL), vortexed for 30 seconds, and centrifuged again under the same conditions. These secondary supernatants were added to the respective EMR-Lipid cartridges and eluted at ∼1 drop/sec, then pooled with the initial eluates. The cartridges were rinsed once more with 0.2 mL of water:acetonitrile (1:4, v/v), followed by a gradual increase in pressure (up to 10 mm Hg) to ensure maximum analyte recovery. Combined eluates were dried under a gentle stream of N_2_ (2 mm Hg, 1 hour) and reconstituted in 0.1 mL of methanol. The reconstituted extracts were transferred into 0.2 mL deactivated glass inserts that were placed inside 2 mL amber glass vials (Agilent Technologies) for LC-MS/MS analysis.

### 2.8 Precision, accuracy, and recovery

To assess method performance, 3 concentrations of quality control (QC) samples (5 ng/mL, low; 50 ng/mL, mid; and 200 ng/mL, high) were prepared in plasma and brain homogenates obtained from naïve CD-1 mice. Each concentration was prepared in triplicate under 2 conditions: pre-EMR (Set A), after protein precipitation without EMR, and post-EMR (Set B), after protein precipitation with EMR cleanup. Each QC sample was run in triplicate across 3 consecutive days alongside calibration standards to assess interday and intraday accuracy and precision. Precision was evaluated by calculating the percent relative SD (% RSD) of replicate measurements for each concentration within a single day. Accuracy was determined as the percent relative error from the nominal concentration, calculated as follows: [(measured concentration)/(nominal concentration)] × 100. Recovery was calculated to evaluate the efficiency of the EMR cleanup process using the formula: (Set B/Set A) × 100.

### 2.9 Matrix effects

Matrix effects were studied using a postcolumn infusion method as previously described.^18^ A 10 *μ*M solution of CBG, prepared in methanol containing 0.1% formic acid, was continuously infused into the mass spectrometric detector source at a flow rate of 0.3 mL/h via a syringe pump. This solution was delivered through a T-connector that was connected with the postcolumn eluent from the LC system. Baseline responses were monitored using selected reaction monitoring transitions. After achieving a steady-state signal, 2.0 *μ*L of either methanol (MeOH blank), pre-EMR brain or plasma extracts (prepared by protein precipitation without EMR cleanup), or post-EMR brain or plasma extracts (prepared by protein precipitation followed by EMR fractionation) was injected into the LC column. LC data were acquired for each injection condition. By overlaying the selected reaction monitoring traces for the MeOH blank, pre-EMR, and post-EMR samples with the total ion chromatograms of CBG and cyclo-CBG, regions exhibiting matrix-induced ion suppression or enhancement were identified.

### 2.10 Method application

The LC-MS/MS method was applied to obtain the PK profile of CBG in plasma and brain tissue of male CD-1 mice. CBG was dissolved in a vehicle consisting of DMSO/Tween-80/saline solution (5:5:90, v/v) and administered at 10 mg/kg (i.p.), using an injection volume of 10 mL/kg. At predetermined time points (15, 30, 60, 120, 240, and 480-minute after injection), mice (*n* = 3–5 per time point) were anesthetized with isoflurane. Blood samples were collected via cardiac puncture using EDTA-rinsed syringes and transferred into 1-mL polypropylene tubes containing spray-coated potassium-EDTA. Plasma was separated by centrifugation (1450 × *g*, 4 °C, 15 minutes) and stored in polypropylene tubes. After blood collection, mice were decapitated, their brains were rapidly dissected on an ice-cold glass plate and flash-frozen on dry ice. All tissue samples were stored at −80 °C until analysis.

### 2.11 Elevated plus maze

The EPM test was conducted under low-intensity lighting (∼10 lux) following established protocols.^19^, ^20^, ^21^ Mice were acclimated to the testing environment in their home cages for at least 1 hour before testing. Each mouse was placed at the center of the maze, facing an open arm opposite the experimenter, and allowed to explore freely for 5 minutes. Exploratory behavior was recorded using an overhead video camera. After each trial, the mouse was returned to its home cage, and the maze was cleaned with SCOE 1× solution (BioFog Inc) and allowed to dry completely to eliminate olfactory cues. A trained observer blinded to treatment conditions manually scored the videos to assess anxiety-like behavior. Quantified measures included the time spent in open and closed arms, the number of entries into each arm type (defined as all 4 paws entering an arm), the number of stretch-attend postures classified as protected (initiated from closed arms or the center) and unprotected (initiated from open arms), and the number of head dips, also classified as protected (performed from closed arms or center) and unprotected (performed from open arms). An anxiety index was calculated for each mouse using the formula^22^: Anxiety Index = 1 − [(time in open arms / total time) + (open arm entries / total entries)] / 2.

### 2.12 Equilibrium dialysis

Equilibrium dialysis coupled with LC-MS/MS was used to determine the unbound fraction (fᵤ) of CBG and cyclo-CBG in mouse plasma and brain homogenate. Mouse brain tissue was homogenized in PBS (pH 7.4) at a 1:4 (w/v) ratio. Plasma and brain homogenate samples were spiked with CBG or cyclo-CBG by adding working solutions (1 *μ*g/mL in DMSO) to achieve final nominal concentrations of 200 and 400 ng/mL, respectively. The final DMSO content was maintained at ≤1% (v/v) in all samples. Equilibrium dialysis was performed using Slide-A-Lyzer devices equipped with 10 kDa Molecular Weight Cut-Off membranes (Thermo Fisher Scientific). Membranes were leak-tested and prerinsed with PBS before use. A 0.2-mL aliquot of spiked plasma or brain homogenate was loaded into the sample chamber and dialyzed against 1.45 mL of PBS (pH, 7.4) in an orbital shaking incubator (INFORS HT) at 37 °C and 150 rpm for 6 hours to ensure equilibrium. Parallel nondialyzed samples (0.2 mL) were incubated under identical conditions to serve as total-concentration controls. After incubation, 100-*μ*L aliquots were collected from both dialyzed samples and nondialyzed controls and stored at −80 °C until analysis. CBG and cyclo-CBG concentrations were quantified using our validated LC-MS/MS method. The fᵤ was calculated as: $f_{u}=\frac{C_{\text{buffer}}}{C_{\text{sample}\;\left(\text{dialyzed}\right)}}$ where *C*_*sample(dialyzed)*_ is the analyte concentration in the postdialysis plasma or brain sample. *C*_*buffer*_, representing the analyte concentration in the postdialysis PBS compartment, was derived from the mass balance relationship: $C_{\text{buffer}}=C_{\text{sample}\;\left(\text{nondialyzed}\right)}-C_{\text{sample}\;\left(\text{dialyzed}\right)}\text{.}$ The unbound brain-to-plasma partition coefficient ($K_{pu,u}$) was calculated as: $K_{pu,u}=\left(\frac{{AUC}_{\text{brain}}}{{AUC}_{\text{plasma}}}\right)\times \left(\frac{f_{u,\text{plasma}}}{f_{u,\text{brain}}}\right)$. All experiments were conducted in triplicate.

### 2.13 Statistical analysis

Pharmacokinetic (PK) parameters, including maximum concentration (C_max_), area under the curve (AUC), elimination half-life (t_1/2_) and brain-to-plasma AUC ratios were determined using a noncompartmental model.^16^^,^^23^ Brain-to-plasma AUC ratios were derived by dividing the AUC values for brain concentrations by the corresponding plasma AUC values over the same time interval. Time to reach *C*_max_ (T_max_) was determined by visual inspection of the mean concentration-time profiles. Behavioral data from the EPM test were analyzed using one-way ANOVA, followed by Šídák’s post hoc test for multiple comparisons. All data are expressed as mean ± SEM, and statistical significance was defined as *P* < .05.

## 3 Results

### 3.1 Analyte identification and quantification

**Fig. 1:**
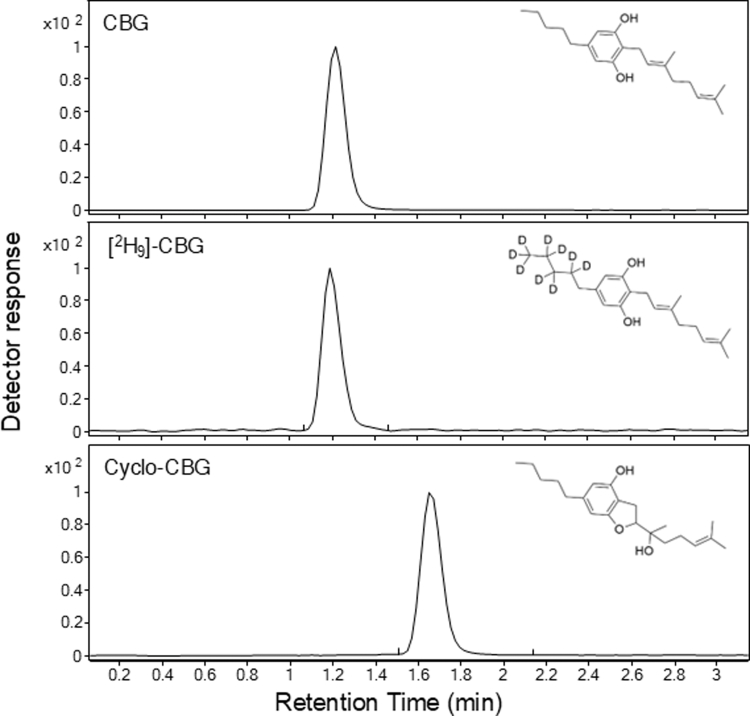
Representative extracted ion chromatograms for CBG (top), internal standard [^2^H_9_]-CBG-CBG (middle), and cyclo-CBG (bottom).

Fragmentation patterns for CBG, cyclo-CBG, and the internal standard [^2^H_9_]-CBG are shown in Supplemental Fig. 1. Notably, CBG and cyclo-CBG shared similar fragmentation patterns and product ions. Their chromatographic properties were, however, readily distinguishable (Fig. 1) and highly reproducible. As shown in Table 2, CBG and cyclo-CBG exhibited highly consistent retention time values of 1.215 ± 0.94 minutes (mean ± SD) and 1.654 ± 1.10 minutes, respectively, with a total runtime of 3.5 minutes. The resolution between the 2 analytes was 2.7, reflecting greater than baseline separation. Capacity factors (k) were 3.4 for CBG and 4.9 for cyclo-CBG, suggesting appropriate column retention. Tailing factors ranged from 1.1 to 1.2 across analytes, indicating symmetrical peaks. Minimal analyte carryover was observed between runs (<0.3%), and no interfering peaks were detected in extracts of mouse plasma or brain at the analytes’ retention time, indicating high analytical specificity.

**Table 2:** Reproducibility of LC-MS/MS replicate runs

Peak	RT (min), *n* = 15	CBG/Cyclo-CBG Resolution	*K*, *n* = 15	Tailing Factor
CBG	1.215 ± 0.296	–	3.4 ± 0.296	1.1
Cyclo-CBG	1.654 ± 0.857	2.7	4.9 ± 0.857	1.2
[^2^H_9_]-CBG	1.188 ± 0.42	–	3.3 ± 0.42	1.2

RT, retention time.

Supplemental Fig. 2 shows representative 15-point calibration curves for CBG and cyclo-CBG. Curve linearity over the full concentration range (0.02–1000 ng/mL) was assessed using a 1/× weighting factor in the absence of matrix. Both analytes exhibited excellent linearity, with correlation coefficients (*R*^2^) of 0.9996 for CBG and 0.9992 for cyclo-CBG (Table 3), a range appropriate for quantifying both compounds in plasma and brain tissue. The lowest limit of detection, defined by a signal-to-noise ratio ≥3, was 0.05 ng/mL for CBG and 0.2 ng/mL for cyclo-CBG. The lowest limit of quantification, based on a signal-to-noise ratio ≥10, was 0.1 ng/mL for CBG and 0.5 ng/mL for cyclo-CBG. The qualifier-to-quantifier ion ratios for both CBG and cyclo-CBG remained >20% and were consistent even at lowest limits of detection and quantification levels.

**Table 3:** Calibration curve parameters for CBG and cyclo-CBG quantification

Analyte	1/× Weighted *R*^2^	LLOD (ng/mL)	fmol/Injection^a^	LLOQ (ng/mL)	fmol/Injection^a^
CBG	0.9996	0.05	0.160	0.1	0.32
Cyclo-CBG	0.9992	0.2	0.602	0.5	1.51

Lower limits of detection (LLOD) and quantification (LLOQ) were determined using signal-to-noise ratios of ≥3 and ≥10, respectively.

a On column concentration based on 1.0 *μ*L injection volume.

### 3.2 Precision, accuracy, and recovery

The intraday and interday accuracy and precision of CBG and cyclo-CBG quantification were evaluated in mouse plasma and brain homogenates, using QC samples spiked either before or after EMR fractionation. Low (5 ng/mL), medium (50 ng/mL), and high (200 ng/mL) QC levels were analyzed in triplicate over 3 consecutive days. Precision is reported as % RSD, and accuracy as the percent deviation from nominal concentrations. In brain homogenates spiked pre-EMR (Table 4A), accuracy ranged from 82.27%–103.41% for CBG and 87.32%–107.8% for cyclo-CBG, with precision (% RSD) between 0.01%–1.52% and 0.31%–7.08%, respectively—fully compliant with Food and Drug Administration bioanalytical validation criteria.^24^ Post-EMR spiking (Table 4B) improved accuracy (CBG: 88.95%–108.77%; cyclo-CBG: 103.46%–121%) while maintaining acceptable precision (CBG: 2.35%–7.30%; cyclo-CBG: 2.28%–8.46%).

**Table 4:** Intraday and interday accuracy and precision of CBG and cyclo-CBG quantification in plasma

A		Day 1			Day 2			Day 3		
Nominal Quantity	Analyte	Mean ± SD (ng/mL)	% RSD	Accuracy (%)	Mean ± SD (ng/mL)	% RSD	Accuracy (%)	Mean ± SD (ng/mL)	% RSD	Accuracy (%)
5 ng/mL	CBG	5.10 ± 0.05	1.01	102.08	5.17 ± 0.04	0.77	103.41	4.84 ± 0.03	0.69	96.88
	Cyclo-CBG	5.18 ± 0.04	0.69	103.53	5.39 ± 0.08	1.48	107.80	5.05 ± 0.02	0.31	100.94
50 ng/mL	CBG	41.13 ± 0.63	1.52	82.27	42.39 ± 0.49	1.17	84.78	41.62 ± 0.54	1.31	83.24
	Cyclo-CBG	43.66 ± 1.99	4.56	87.32	47.78 ± 2.15	4.49	95.57	45.91 ± 3.25	7.08	91.82
200 ng/mL	CBG	180.83 ± 1.33	0.73	90.42	185.27 ± 0.03	0.01	92.63	182.10 ± 2.23	1.23	91.05
	Cyclo-CBG	194.47 ± 4.87	2.50	97.24	208.28 ± 3.45	1.66	104.14	203.93 ± 4.40	2.16	101.97

B		Day 1			Day 2			Day 3		
Nominal Quantity	Analyte	Mean ± SD (ng/mL)	% RSD	Accuracy (%)	Mean ± SD (ng/mL)	% RSD	Accuracy (%)	Mean ± SD (ng/mL)	% RSD	Accuracy (%)
5 ng/mL	CBG	5.35 ± 0.38	7.11	107.10	5.44 ± 0.40	7.30	108.77	5.14 ± 0.33	6.37	102.86
	Cyclo-CBG	5.82 ± 0.33	5.73	116.47	6.05 ± 0.27	4.43	121.00	5.79 ± 0.43	7.41	115.71
50 ng/mL	CBG	44.48 ± 1.07	2.40	88.95	45.78 ± 1.08	2.35	91.57	44.84 ± 1.49	3.33	89.67
	Cyclo-CBG	51.73 ± 1.18	2.29	103.46	54.44 ± 1.63	2.99	108.87	53.68 ± 1.22	2.28	107.36
200 ng/mL	CBG	190.89 ± 6.60	3.46	95.44	195.02 ± 8.31	4.26	97.51	191.52 ± 10.29	5.37	95.76
	Cyclo-CBG	220.19 ± 17.48	7.94	110.10	235.83 ± 19.96	8.46	117.91	226.33 ± 18.61	8.22	113.16

Standards were spiked either (A) before or (B) after EMR fractionation. Analytes were analyzed over 3 consecutive days.

In plasma (Table 5), post-EMR spiking produced acceptable accuracy and precision at all concentrations. For pre-EMR spiked plasma samples, accuracy met Food and Drug Administration criteria at 5 and 200 ng/mL but was slightly below the acceptable range at 50 ng/mL (CBG: 79.59%–81.44%; cyclo-CBG: 86.62%–88.55%). Nonetheless, precision remained within acceptable limits across all levels (CBG: 0.49%–11.46% RSD; cyclo-CBG: 0.05%–11.15% RSD).

**Table 5:** Intraday and interday accuracy and precision of CBG and cyclo-CBG quantification in brain tissue

A		Day 1			Day 2			Day 3		
Nominal Quantity	Analyte	Mean ± SD (ng/mL)	% RSD	Accuracy (%)	Mean ± SD (ng/mL)	% RSD	Accuracy (%)	Mean ± SD (ng/mL)	% RSD	Accuracy (%)
5 ng/mL	CBG	5.15 ± 0.02	0.49	102.93	5.26 ± 0.07	1.30	105.15	5.02 ± 0.10	1.94	100.38
	Cyclo-CBG	5.58 ± 0.14	2.46	111.56	5.66 ± 0.00	0.05	113.25	5.40 ± 0.14	2.64	108.05
50 ng/mL	CBG	39.80 ± 3.80	9.54	79.59	40.72 ± 4.15	10.19	81.44	40.03 ± 4.03	10.07	80.06
	Cyclo-CBG	43.31 ± 4.83	11.15	86.62	44.16 ± 4.61	10.45	88.32	44.28 ± 4.68	10.56	88.55
200 ng/mL	CBG	195.60 ± 22.42	11.46	97.80	201.02 ± 22.48	11.18	100.51	196.20 ± 22.04	11.24	98.10
	Cyclo-CBG	217.78 ± 21.98	10.09	108.89	229.47 ± 19.38	8.44	114.74	224.95 ± 22.23	9.88	112.47

B		Day 1			Day 2			Day 3		
Nominal Quantity	Analyte	Mean ± SD (ng/mL)	% RSD	Accuracy (%)	Mean ± SD (ng/mL)	% RSD	Accuracy (%)	Mean ± SD (ng/mL)	% RSD	Accuracy (%)
5 ng/mL	CBG	5.40 ± 0.10	1.78	108.10	5.57 ± 0.07	1.23	111.40	5.25 ± 0.08	1.55	105.00
	Cyclo-CBG	5.79 ± 0.17	3.00	115.85	6.06 ± 0.24	3.98	121.10	5.66 ± 0.12	2.16	113.16
50 ng/mL	CBG	45.62 ± 1.51	3.31	91.24	46.13 ± 1.80	3.90	92.26	45.39 ± 2.15	4.73	90.79
	Cyclo-CBG	49.46 ± 1.57	3.17	98.93	50.40 ± 1.55	3.08	100.79	50.42 ± 1.52	3.01	100.85
200 ng/mL	CBG	184.13 ± 7.24	3.93	92.06	191.14 ± 7.60	3.98	95.57	183.68 ± 6.44	3.50	91.84
	Cyclo-CBG	199.69 ± 6.83	3.42	99.84	215.06 ± 9.77	4.54	107.53	211.23 ± 11.60	5.49	105.62

Standards were spiked either (A) before or (B) after EMR fractionation. Analytes were analyzed over 3 consecutive days.

Analyte recovery, calculated as (pre-EMR response/post-EMR response) × 100, was robust across matrixes (Table 6). In plasma, recovery ranged from 87.5%–107.3%, reflecting efficient extraction and occasional matrix-enhanced ionization. In brain, recovery values ranged from 85.9%–95.3%, indicating consistent extraction performance with minimal matrix interference.

**Table 6:** Recovery of CBG and cyclo-CBG from plasma and brain tissue after EMR fractionation

Nominal quantity	Analyte	Plasma	Brain
		Recovery (%)	Recovery (%)
5 ng/mL	CBG	95.1	95.3
	Cyclo-CBG	95.2	88.7
50 ng/mL	CBG	87.7	92.7
	Cyclo-CBG	87.5	85.9
200 ng/mL	CBG	106.0	95.1
	Cyclo-CBG	107.3	89.7

Recovery (%) was calculated as (pre-EMR response/post-EMR response) × 100, where pre-EMR response is the mean peak area of analytes spiked before EMR processing, and post-EMR response is the mean peak area of the same amount spiked after EMR (*n* = 3 samples per condition, run in duplicate).

### 3.3 Matrix effects

**Fig. 2:**
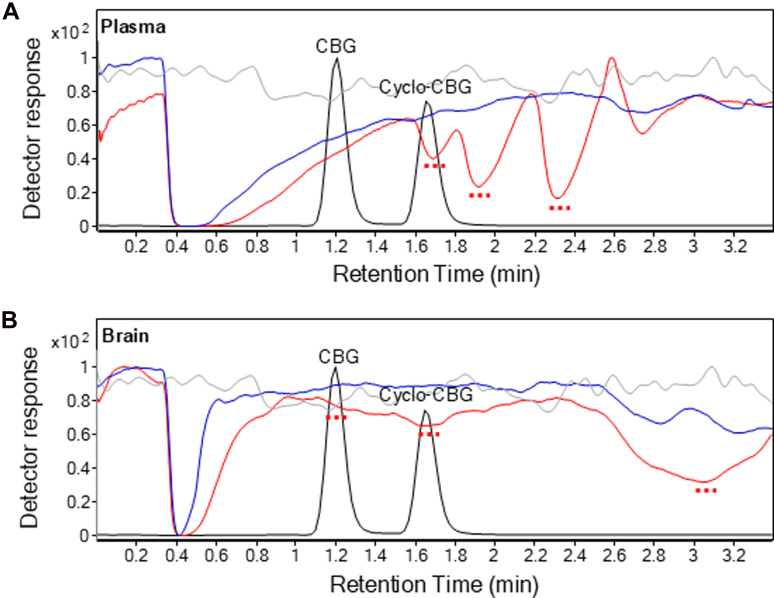
Matrix effects for CBG (Multiple Reaction Monitoring transition *m/z* 317.3 > 193.0) in mouse plasma (A) and brain tissue extracts (B), before and after EMR fractionation. Chromatograms show matrix background signals from pre-EMR (red), post-EMR (blue), and methanol control injections (gray). Red dotted lines indicate regions of signal suppression. Black, overlaid LC tracing shows the elution of CBG and cyclo-CBG.

Matrix effects were evaluated in mouse plasma and brain homogenates using postcolumn infusion and pre-extraction and postextraction spikes of matrixes.^18^^,^^25^ In postcolumn infusion, analytes were continuously delivered while injecting matrix extracts to detect real-time signal alterations due to ion suppression or enhancement. As shown in Fig. 2A, pre-EMR plasma matrix injections (red trace) resulted in pronounced ion suppression within the retention windows of CBG (*m/z* 317.3 > 193.0) and cyclo-CBG (*m/z* 333.3 > 193.0), relative to methanol controls (gray trace). In contrast, pre-EMR brain samples exhibited milder suppression (Fig. 2B). Importantly, EMR cleanup substantially reduced these effects in both plasma and brain matrixes (blue trace), indicating effective mitigation of matrix interference. These findings confirm that EMR markedly improves matrix-related ion suppression, enhancing the specificity and reliability of analyte quantification.

### 3.4 Plasma and brain pharmacokinetics

**Fig. 3:**
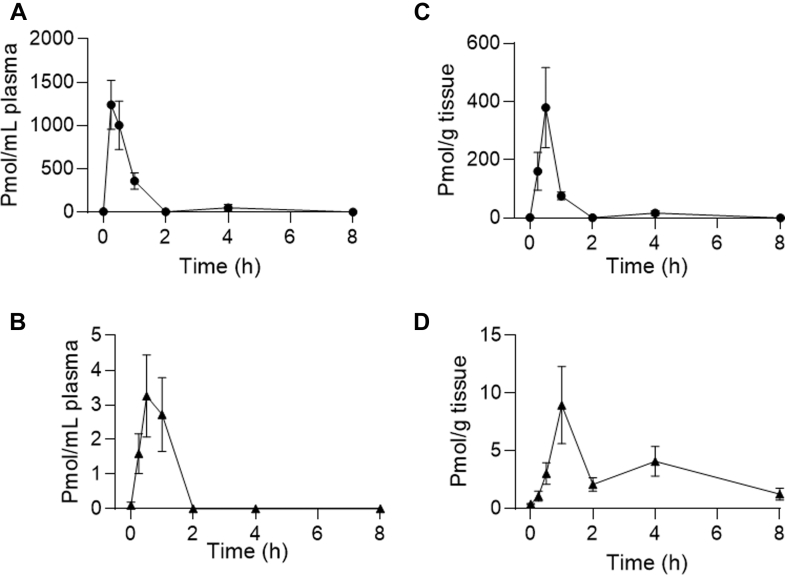
Time course of plasma (A, C) and brain (B, D) concentrations of CBG (black circles) and cyclo-CBG (black triangles) after a single intraperitoneal injection of CBG (10 mg/kg). Data are presented as mean ± SEM, with 3 to 5 animals per time point.

Plasma and brain concentrations of CBG and cyclo-CBG were quantified after a single intraperitoneal injection of CBG (10 mg/kg) in adult male CD-1 mice. Samples were collected at 15, 30, 60,120, 240, and 480 minutes after injection (*n* = 3–5 per time point). In plasma, CBG achieved a peak concentration (C_max_) of 1240.25 ± 281.60 pmol/mL at 15 minutes (T_max_) after administration, subsequently declining with a half-life (t_1/2_) of 45 minutes (Fig. 3A; Table 7). Cyclo-CBG plasma concentrations peaked at 30 minutes, with a C_max_ of 3.25 ± 1.19 pmol/mL (Fig. 3B; Table 7). Across the 8-hour time course, the plasma concentrations of cyclo-CBG remained over 400-fold lower than CBG’s, suggesting either limited oxidative metabolism or rapid metabolite clearance.

**Table 7:** Pharmacokinetic parameters of CBG and cyclo-CBG in mouse plasma and brain tissue

			Plasma			Brain		
Analyte	C_max_ (pmol/mL)	AUC (pmol•min/mL)	T_max_ (min)	t_1/2_ (min)	C_max_ (pmol/mL)	AUC (pmol•min/mL)	T_max_ (min)	t_1/2_ (min)
CBG	1240.25 ± 281.60	66,875 ± 14,868	15	45.65 ± 2.10	379.9 ± 137.71	17,609 ± 5813	30	57.02 ± 8.04
Cyclo-CBG	3.25 ± 1.19	220.1 ± 80.08	30	–	8.96 ± 3.33	1565 ± 402.1	60	–

Parameters include maximal concentration (C_max_), AUC, time to reach C_max_ (T_max_), and half-life of elimination (t_1/2_). Data are presented as mean ± SD.

**Fig. 4:**
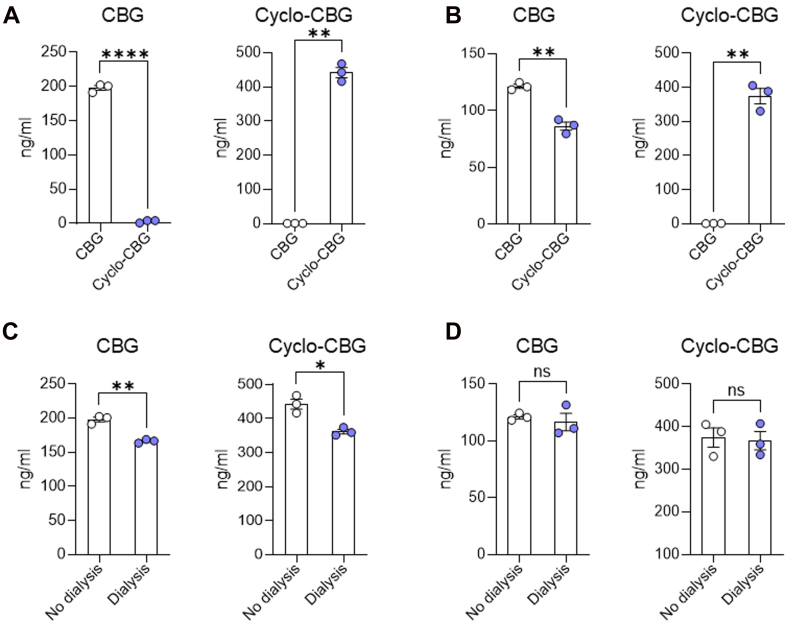
Ex vivo oxidative conversion of CBG to cyclo-CBG in mouse plasma (A) and brain homogenate (B). Panels (C and D) show CBG and cyclo-CBG concentrations measured in dialyzed and total (nondialyzed) samples from mouse plasma (C) and brain homogenate (D) after equilibrium dialysis. Data are presented as mean ± SEM (*n* = 3/group). Statistical comparisons were performed using unpaired *t* tests with Welch’s correction. ∗*P* < .05, ∗∗*P* < .01, and ∗∗∗∗*P* < .0001; ns, not significant.

In brain tissue, CBG reached a C_max_ of 379.90 ± 137.71 pmol/g at 30 minutes after injection (Fig. 3C; Table 7), suggesting a rapid transfer through the blood–brain barrier. CBG levels declined with a t_1/2_ of 57 minutes. Brain cyclo-CBG concentrations peaked at 60 minutes, attaining a C_max_ of 8.96 ± 3.33 pmol/g (Fig. 3D). The time course of cyclo-CBG accumulation along with the unexpectedly high brain levels attained by this metabolite—compared with plasma—suggest either local oxidative metabolism of CBG or preferential cyclo-CBG retention. To test this hypothesis, we conducted complementary ex vivo experiments in which mouse plasma and brain homogenate were spiked with CBG (200 ng/mL) and incubated at 37 °C for 6 hours under orbital agitation. As shown in Fig. 4, CBG underwent low but measurable conversion to cyclo-CBG in plasma (3.08 ± 1.99 ng/mL). In contrast, conversion in brain homogenate was markedly greater, yielding 86.14 ± 6.22 ng/mL cyclo-CBG, consistent with the occurrence of oxidative metabolism in brain tissue. Interestingly, both CBG and cyclo-CBG exhibited a modest secondary rise in brain concentrations at 240 minutes, with CBG increasing to 17.22 ± 8.57 pmol/g and cyclo-CBG to 4.09 ± 1.28 pmol/g (Fig. 3, C and D; Table 7). This biphasic pattern may reflect redistribution from peripheral compartments or delayed central nervous system equilibration. To quantify brain penetration, brain-to-plasma AUC_0–480 min_ ratios were calculated. CBG and cyclo-CBG exhibited brain-to-plasma AUC ratios of 0.26 and 7.1, respectively, further pointing to local oxidative metabolism of CBG in brain tissue.

### 3.5 Calculation of K_p_ᵤ,ᵤ

Table 8 summarizes the concentrations of CBG and cyclo-CBG measured in the dialyzed samples, nondialyzed samples, and PBS buffer, along with the calculated fᵤ values. In plasma, CBG exhibited a *C*_*buffer*_ of 31.7 ± 6.68 ng/mL and a dialyzed sample concentration of 166.4 ± 2.77 ng/mL. The resulting unbound fraction was fᵤ = 0.19 ± 0.04, indicating that approximately 19% of CBG is unbound in mouse plasma. Cyclo-CBG showed a higher *C*_*buffer*_ (80.9 ± 28.31 ng/mL) and a dialyzed plasma concentration of 361.6 ± 11.63 ng/mL, corresponding to an fᵤ of 0.224 ± 0.082 (∼22% unbound). In brain homogenate, both analytes displayed substantially lower unbound fractions. For CBG, the *C*_*buffer*_ was 4.60 ± 13.55 ng/mL and a dialyzed brain concentration of 116.70 ± 13.17 ng/mL, resulting in an fᵤ of 0.039 ± 0.12. Cyclo-CBG exhibited a *C*_*buffer*_ of 10.7 ± 54.11 ng/mL and a dialyzed brain concentration of 367.0 ± 37.17 ng/mL, yielding an fᵤ of 0.02 ± 0.15. These fᵤ values were used to calculate the unbound brain-to-plasma partition coefficient (*K*_*p*_*ᵤ,ᵤ*). For CBG, *K*_*p*_*ᵤ,ᵤ* was approximately 1.26, indicating near-equilibration of unbound drug across the blood–brain barrier with only modest enrichment in brain. In contrast, cyclo-CBG showed a markedly elevated *K*_*p*_*ᵤ,ᵤ* of ∼75.7, reflecting a strong preferential accumulation of unbound drug within the central nervous system after correction for binding differences.

**Table 8:** Concentrations in PBS buffer, dialyzed samples, total samples, and corresponding unbound fractions (fᵤ) for CBG and Cyclo-CBG in mouse plasma and brain homogenate after equilibrium dialysis

			Plasma			Brain		
Analyte	C_buffer_ (ng/mL)	C_sample(dialyzed)_ (ng/mL)	C_sample(nondialyzed)_ (ng/mL)	f_u_	C_buffer_ (ng/mL)	C_sample(dialyzed)_ (ng/mL)	C_sample(nondialyzed)_ (ng/mL)	f_u_
CBG	31.7 ± 6.68	166.4 ± 2.768	198.1 ± 6.083	0.191	4.6 ± 13.55	116.7 ± 13.17	121.3 ± 3.203	0.039
Cyclo-CBG	80.9 ± 28.31	361.6 ± 11.63	442.5 ± 25.81	0.224	10.7 ± 54.11	367.0 ± 37.17	374.7 ± 39.32	0.021

Data are expressed as mean ± SD (*n* = 3 samples per condition, run in duplicate). As defined, fᵤ was obtained by dividing the analyte concentration in buffer by that in the matched dialyzed sample.

### 3.6 Effects of CBG in the EPM

**Fig. 5:**
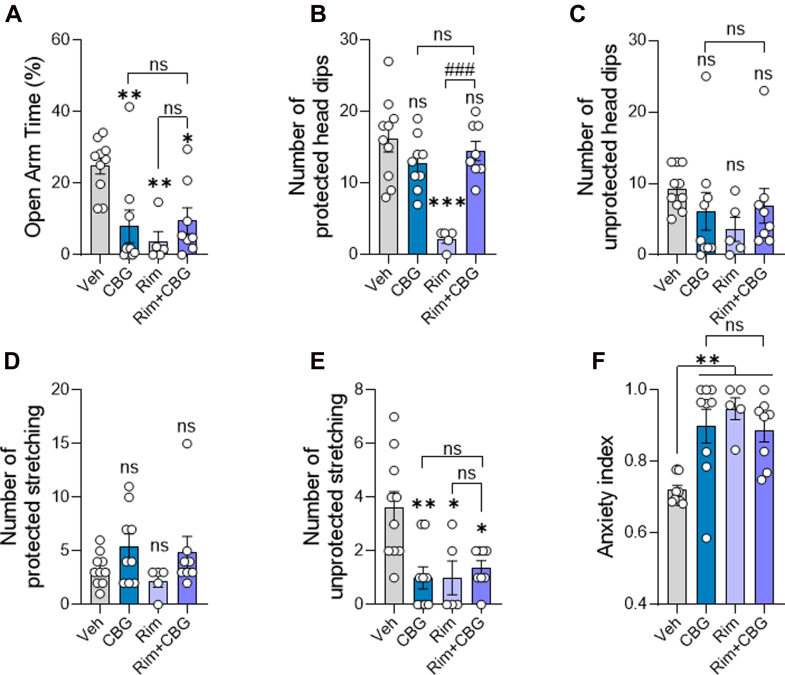
Acute CBG administration increases anxiety-like behavior in the EPM test. Mice received vehicle (Veh), CBG (10 mg/kg), rimonabant (Rim; 10 mg/kg), or Rim 30 minutes before CBG (Rim + CBG). EPM was conducted 30 minutes after CBG (or 60 minutes after Rim alone). (A) CBG reduced % open arm time. (B–D) No changes were observed in protected head dips, unprotected head dips, or protected stretch-attend postures. (E) CBG reduced unprotected stretch-attend postures. (F) CBG and/or Rim increased the anxiety index. Data are shown as mean ± SEM (*n* = 5–10/group) and analyzed by one-way ANOVA with Šídák’s post hoc test. ∗*P* < .05, ∗∗*P* < .01, ∗∗∗*P* < .001 vs Veh; ^###^*P* < .001 vs Rim; ns, not significant.

The rapid appearance of significant amounts of CBG and cyclo-CBG in the brain prompted us to examine potential functional consequences. Given prior reports of anxiolytic effects,^5^^,^^10^ we administered CBG at the previously characterized 10 mg/kg dose and assessed anxiety-like behavior in the EPM, 30 minutes after injection, corresponding to the time of peak brain concentrations. Rimonabant (SR14716A, 10 mg/kg, i.p.), a CB_1_ receptor inverse agonist known to induce anxiety-like behavior,^26^ was used as a positive control and to assess whether the effects of CBG might involve CB_1_ signaling. Consistent with its known anxiogenic profile,^26^ rimonabant significantly reduced the percentage of time spent in the open arms compared with vehicle controls (Fig. 5A), decreased the number of protected (but not unprotected) head dips (Fig. 5, B and C), decreased the number of unprotected (but not protected) stretch-attend postures (Fig. 5, D and E), and increased the anxiety index (Fig. 5F). In contrast to previous reports, CBG-treated mice also exhibited increased anxiety-like behavior. Specifically, CBG significantly reduced open arm time relative to vehicle (Veh vs CBG: Δ_mean_ = 16.96%; 95% CI, 3.708–30.21; *P* = .0069; Fig. 5A), decreased unprotected stretch-attend postures (Δ_mean_ = 2.600; 95% CI, 0.724–4.47; *P* = .003; Fig. 5E), and elevated the anxiety index (Δ_mean_ = −0.178; 95% CI, −0.302 to −0.0546; *P* = .002; Fig. 5F). These anxiogenic-like effects were not attributable to motor suppression since the number of entries in closed arm, an index of locomotor activity, did not differ significantly between groups (Supplemental Fig. 3). Importantly, pretreatment with rimonabant neither attenuated nor enhanced the anxiogenic-like effects of CBG. No significant differences were observed between the CBG and CBG plus rimonabant groups across all 3 measures (eg, open arm time: Δ_mean_ = −1.499%; 95% CI: −15.51 to 12.52; *P* = .9998). Together, the findings suggest that CBG exerts anxiogenic effects in male mice, which do not involve CB_1_ receptor signaling.

## 4 Discussion

This paper describes a fast, sensitive, and reproducible LC-MS/MS method for quantifying CBG and its primary oxidative metabolite, cyclo-CBG,^14^ in biological samples. By incorporating EMR fractionation during sample preparation, we minimized matrix effects and improved analyte recovery. CBG and cyclo-CBG were chromatographically well resolved, with consistent retention times, minimal carryover, and adequate limits of detection and quantification. The method met Food and Drug Administration criteria for accuracy, precision, and recovery, supporting its utility in PK studies in preclinical and clinical studies. Using this platform, we characterized the PK profile of CBG after intraperitoneal administration in male CD-1 mice. The agent distributed rapidly into plasma and brain but was also quickly cleared from both compartments. Despite its high lipophilicity (calculated LogP ∼6.44),^27^ CBG exhibited a relatively low brain-to-plasma ratio (0.26). Active transporter-mediated efflux mechanisms at the blood–brain barrier may, at least partially, contribute to its restricted central nervous system exposure in vivo. Consistent with this possibility, CBG has been reported to be a weak substrate of the breast cancer resistance protein (BCRP/ABCG2) transporter, whereas not a significant substrate of the major efflux transporter P-glycoprotein (P-gp/ABCB1).^28^

In striking contrast, cyclo-CBG exhibited a surprisingly high brain-to-plasma ratio (7.1), indicating substantial brain exposure to this bioactive metabolite. The magnitude of cyclo-CBG brain accumulation raises the possibility that CBG undergoes local metabolic conversion within brain tissue, where multiple cytochrome P450 enzymes are known to be expressed.^29^ Several P450 isoforms implicated in the oxidative cyclization of CBG in human hepatocytes (CYP2J2, CYP3A4, CYP2C8, CYP2C9, CYP2B6, CYP2C19, and CYP2D6) have identifiable orthologs in mouse brain.^14^^,^^30^ For example, CYP2J9, the murine ortholog of CYP2J2, is abundant in cerebellar Purkinje cells,^31^ and CYP3A11 and CYP3A13—the murine orthologs of CYP3A4—are expressed in neurons.^32^ Consistent with this enzymatic landscape, our in vitro studies demonstrated detectable conversion of CBG to cyclo-CBG in brain homogenate and, to a much lesser extent, in plasma. These findings together support the plausibility of local, brain-mediated generation of cyclo-CBG.

Equilibrium dialysis experiments further revealed low unbound fractions for both CBG and cyclo-CBG in brain homogenate and disproportionately high *K*_*p*_*ᵤ,ᵤ* values for cyclo-CBG, consistent with extensive tissue sequestration. Such sequestration may amplify brain retention of cyclo-CBG irrespective of the site of formation, although our enzymatic and PK findings together support the possibility that both local generation and preferential neural compartmentalization contribute to its striking brain accumulation.

Despite its commercial availability, CBG remains largely understudied. One of its current uses is as self-medication for anxiety: in an online survey published in 2022, 51.2% of respondents rated CBG-predominant cannabis products as more effective than conventional anxiolytic medications.^3^ This report aligns with 2 preclinical studies reporting anxiolytic-like effects of CBG (10 mg/kg) in mice^4^ and rats.^5^ In humans, a placebo-controlled field trial involving 34 participants found that a single oral dose of 20 mg CBG significantly reduced self-reported anxiety.^10^ However, not all studies support this narrative. In one study, CBG administration in rats (2.5 mg/kg, i.p.) did not significantly affect anxiety-like behavior in the light-dark box test.^33^ Similarly, in a mouse model of posttraumatic stress, CBG at 10 or 30 mg/kg (i.p.) failed to attenuate anxiety-like responses.^34^

To help reconcile these conflicting findings, we tested the acute effects of CBG on anxiety-like behavior in male mice, at a time point aligned with its peak brain levels. Surprisingly, CBG produced anxiogenic-like effects in the EPM test, reducing open arm exploration and increasing anxiety index scores. These effects were not reversed by rimonabant, a CB_1_ inverse agonist, and CBG only partially mimicked the behavioral profile of rimonabant in the EPM test, suggesting that its anxiogenic action does not involve CB_1_ signaling. Given the selective accumulation of cyclo-CBG in the brain, it is plausible that this metabolite contributes to the observed anxiogenic response, although this hypothesis remains to be experimentally verified.

The timing of behavioral assessment may also explain discrepancies in the literature. Here, testing occurred 30 minutes after CBG administration, corresponding to peak brain CBG levels. In contrast, studies reporting anxiolytic-like effects assessed behavior approximately 60 minutes after administration in both mice and rats.^4^^,^^5^ Studies reporting no anxiolytic effect used doses both lower^33^ and higher^34^, ^35^, ^36^ than those used here.

Several limitations warrant consideration. First, all experiments were conducted in adult male mice, and the findings may not generalize to females. Second, although we quantified CBG and its major metabolite cyclo-CBG, other metabolites (eg, 6′,7′-epoxy-CBG) were not measured, leaving their potential contributions unresolved. Third, behavioral testing was performed at only one dose and time point, selected to match peak brain CBG levels. This design does not capture the effects of CBG at other times or doses. Fourth, although cyclo-CBG showed striking brain accumulation, we did not directly assess its behavioral effects, so its role in the observed anxiogenic-like responses remains speculative. Finally, only the intraperitoneal route was examined, and additional routes (eg, oral, intravenous) will be important for translational relevance.

Despite these limitations, the present study provides a robust LC-MS/MS method for quantifying CBG and its oxidative metabolite, cyclo-CBG, in biological matrixes, and delineates key PK properties of CBG in adult male mice, which could aid future research. Our study also reveals an unexpected anxiogenic-like effect of CBG when assessed at peak brain exposure. As interest in CBG continues to grow, these findings highlight the critical need for rigorous PK evaluation and point to the potential role of cyclo-CBG in mediating the central effects of CBG.

## Conflict of interest

The authors declare no conflicts of interest.
