# Supplementary material for: Pharmacokinetic and pharmacodynamic properties of cannabigerol in male mice
Source: J Pharmacol Exp Ther. 2026 Feb 24;393(4):104308. doi: 10.1016/j.jpet.2026.104308 (PMC13197944; doi:10.1016/j.jpet.2026.104308)
Supplement: Supplementary Figures 1-3 [file mmc1.pptx]

## Slide 1
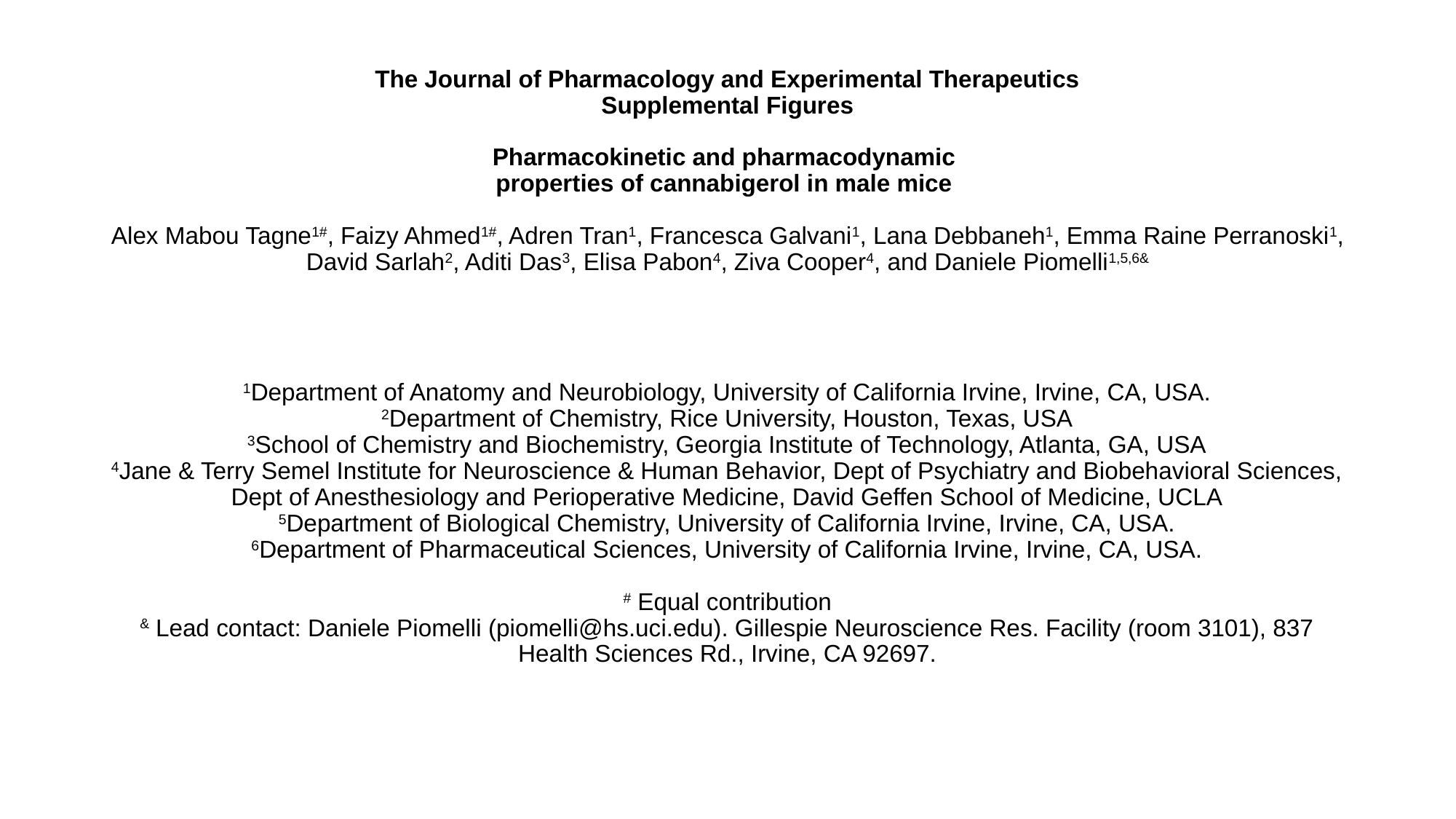

# The Journal of Pharmacology and Experimental Therapeutics Supplemental Figures Pharmacokinetic and pharmacodynamic properties of cannabigerol in male mice   Alex Mabou Tagne1#, Faizy Ahmed1#, Adren Tran1, Francesca Galvani1, Lana Debbaneh1, Emma Raine Perranoski1, David Sarlah2, Aditi Das3, Elisa Pabon4, Ziva Cooper4, and Daniele Piomelli1,5,6& 1Department of Anatomy and Neurobiology, University of California Irvine, Irvine, CA, USA. 2Department of Chemistry, Rice University, Houston, Texas, USA 3School of Chemistry and Biochemistry, Georgia Institute of Technology, Atlanta, GA, USA 4Jane & Terry Semel Institute for Neuroscience & Human Behavior, Dept of Psychiatry and Biobehavioral Sciences, Dept of Anesthesiology and Perioperative Medicine, David Geffen School of Medicine, UCLA 5Department of Biological Chemistry, University of California Irvine, Irvine, CA, USA. 6Department of Pharmaceutical Sciences, University of California Irvine, Irvine, CA, USA. # Equal contribution & Lead contact: Daniele Piomelli (piomelli@hs.uci.edu). Gillespie Neuroscience Res. Facility (room 3101), 837 Health Sciences Rd., Irvine, CA 92697.

## Slide 2
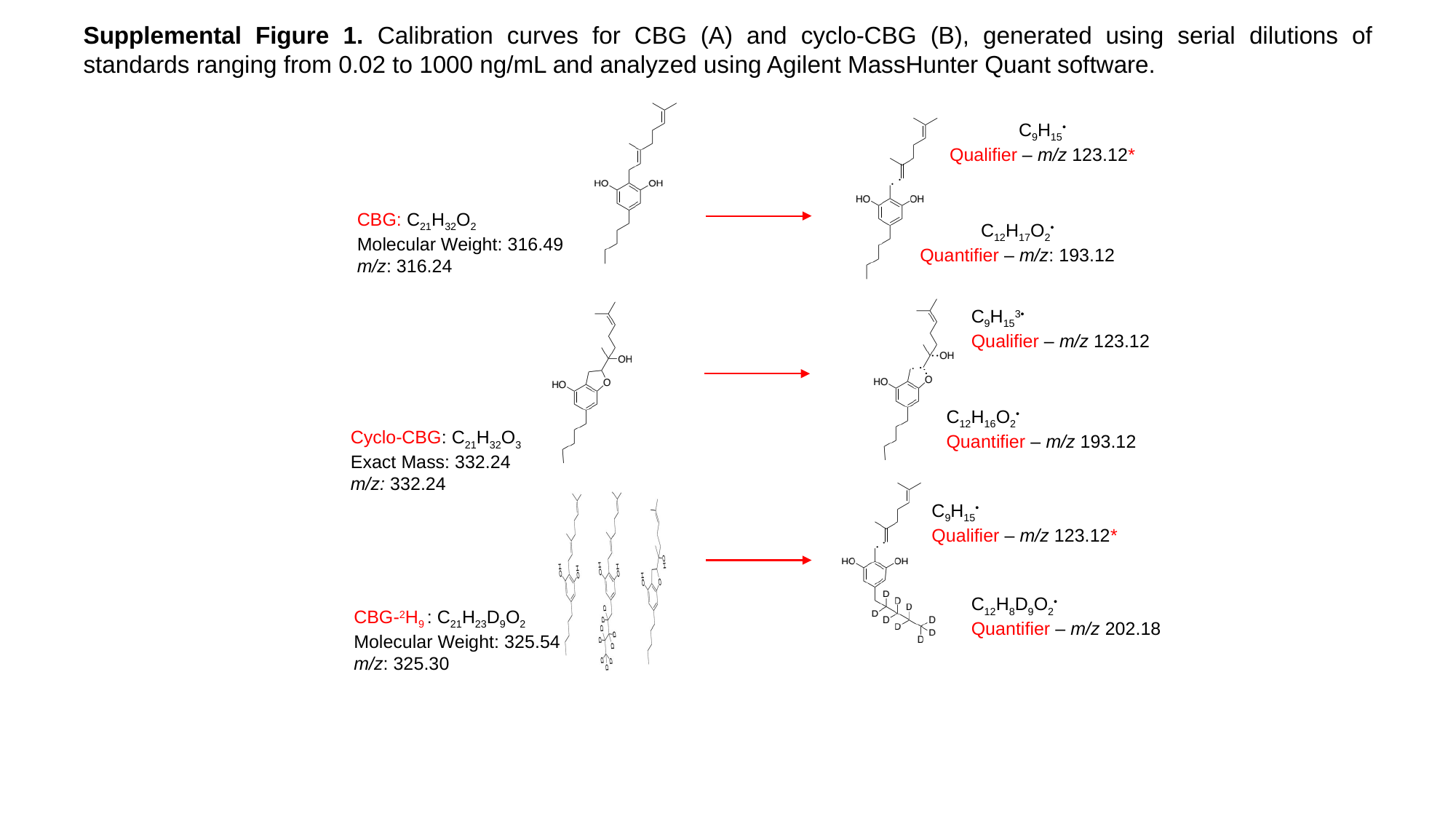

Supplemental Figure 1. Calibration curves for CBG (A) and cyclo-CBG (B), generated using serial dilutions of standards ranging from 0.02 to 1000 ng/mL and analyzed using Agilent MassHunter Quant software.
C9H15•
Qualifier – m/z 123.12*
CBG: C21H32O2
Molecular Weight: 316.49
m/z: 316.24
C12H17O2•
Quantifier – m/z: 193.12
C9H153•
Qualifier – m/z 123.12
C12H16O2•
Quantifier – m/z 193.12
Cyclo-CBG: C21H32O3
Exact Mass: 332.24
m/z: 332.24
C9H15•
Qualifier – m/z 123.12*
C12H8D9O2•
Quantifier – m/z 202.18
CBG-2H9 : C21H23D9O2
Molecular Weight: 325.54
m/z: 325.30

## Slide 3
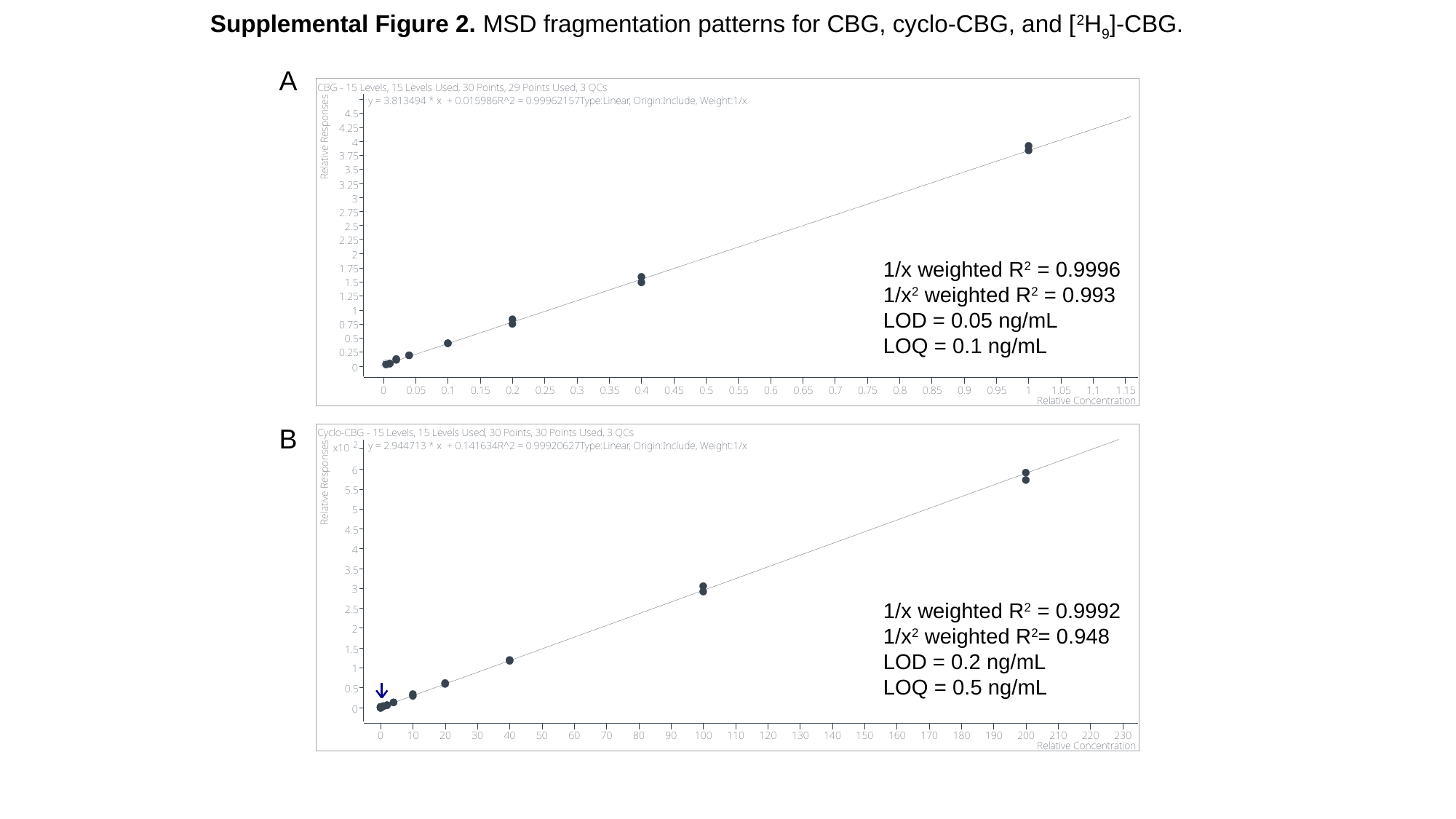

Supplemental Figure 2. MSD fragmentation patterns for CBG, cyclo-CBG, and [2H9]-CBG.
A
1/x weighted R2 = 0.9996
1/x2 weighted R2 = 0.993
LOD = 0.05 ng/mL
LOQ = 0.1 ng/mL
B
1/x weighted R2 = 0.9992
1/x2 weighted R2= 0.948
LOD = 0.2 ng/mL
LOQ = 0.5 ng/mL

## Slide 4
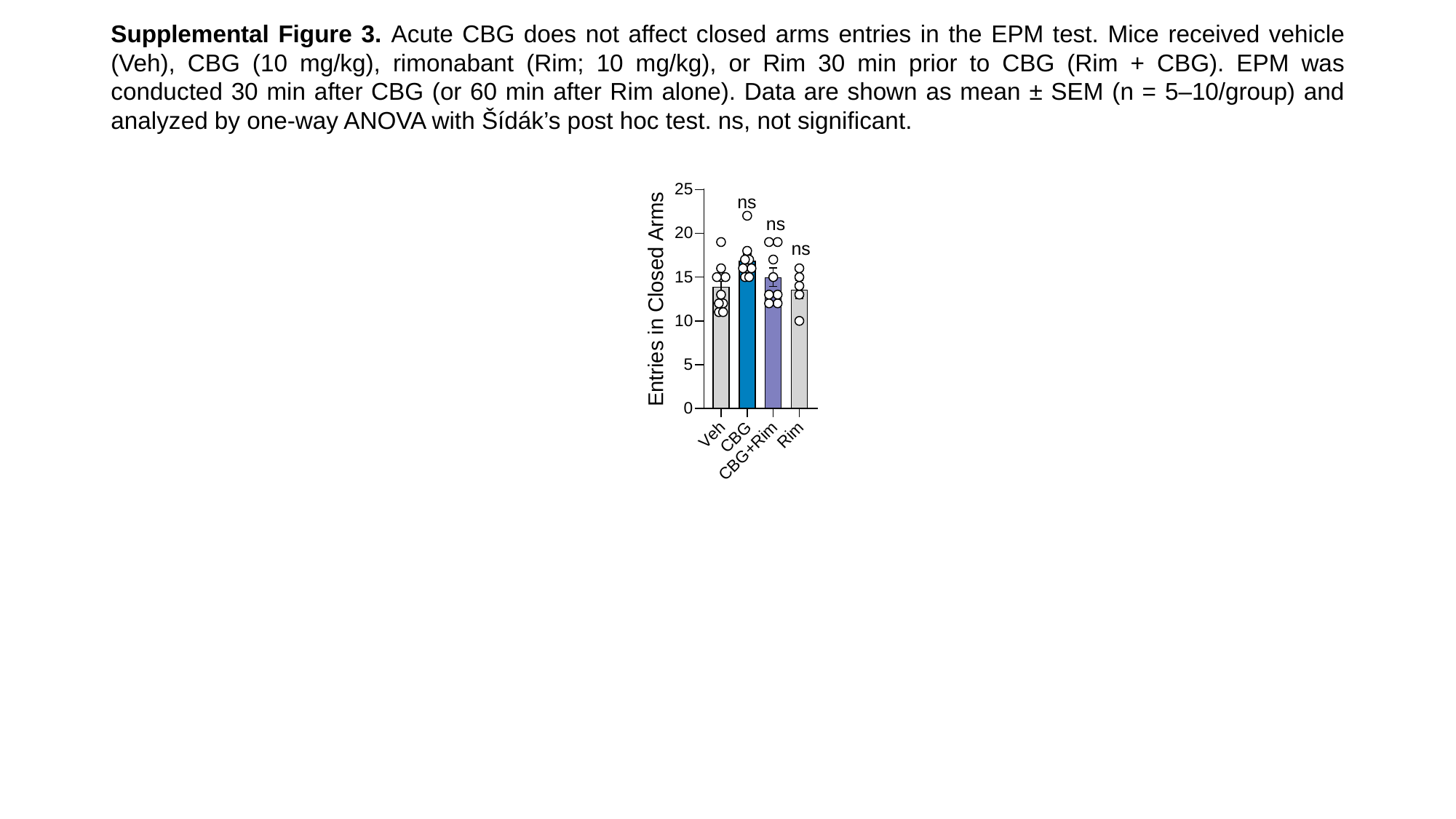

Supplemental Figure 3. Acute CBG does not affect closed arms entries in the EPM test. Mice received vehicle (Veh), CBG (10 mg/kg), rimonabant (Rim; 10 mg/kg), or Rim 30 min prior to CBG (Rim + CBG). EPM was conducted 30 min after CBG (or 60 min after Rim alone). Data are shown as mean ± SEM (n = 5–10/group) and analyzed by one-way ANOVA with Šídák’s post hoc test. ns, not significant.
